# Evaluating the effect of body mass index and 25-hydroxy-vitamin D level on basal cell carcinoma using Mendelian randomization

**DOI:** 10.1038/s41598-023-43926-w

**Published:** 2023-10-02

**Authors:** Xuezhao Chen, Shan Song, Jinyu Shi, Zhiyao Wang, Wenyu Song, Jiaxin Wang, Guoyan Wang, Xiaobing Wang

**Affiliations:** 1 Shanxi Medical University, Taiyuan, China; 2 Department of Plastic Surgery, The First Hospital of Shanxi Medical University, Taiyuan, 030000 Shanxi China; 3 Department of Rheumatology, The Second Hospital of Shanxi Medical University, Taiyuan, China; 4 Department of Breast Surgery, The Fifth Hospital of Shanxi Medical University, Taiyuan, China; 5 Department of Clinical Medical College, Shanxi Datong University, Datong, China

**Keywords:** Cancer, Risk factors

## Abstract

Basal cell carcinoma (BCC) is the most common cancer with a rising incidence among white-skinned individuals. A number of epidemiological studies have suggested that obesity and serum 25-hydroxy-vitamin D (25(OH)D) levels may affect the arising of BCC. To address this, we selected 443 and 96 single nucleotide polymorphisms (SNPs) associated with body mass index (BMI) and serum level of 25(OH)D from large-scale genome-wide association studies (GWAS), respectively. The univariable and multivariable two-sample Mendelian randomization (MR) analyses were conducted with a series of sensitivity analyses to ensure the results were reliable and reproducible. The results of univariable two-sample MR analysis showed that higher BMI was related to lower risk for BCC (Odds ratio(OR) = 0.90; 95% confidence interval (CI),[0.81,0.99]; *p* = 0.02). In addition, this causal effect of BMI on BCC still remained (OR = 0.88; 95%CI,[− 0.22, − 0.03], p-value = 0.008) after adjusting for 25(OH)D level in the multivariable MR analysis. However, the results suggested that 25(OH)D level was not associated with BCC(OR = 1.02; 95%CI, [0.94,1.09], p-value = 0.67). In conclusion, similar to the conclusions of retrospective observational studies, the MR results indicate that high BMI is an independent protective factor for BCC. Meanwhile, vitamin D levels may not be causally associated with the risk of basal cell carcinoma and increasing vitamin D supplementation is unlikely to reduce the risk.

## Introduction

In fair-skinned adults, the most common type of skin cancer is basal cell carcinoma (BCC). Its lifetime risk is estimated to be 30%, and its worldwide incidence is rising rapidly. BCC's high occurrence leads to a significant economic burden for public health finance. Although BCC's etiology remains unclear, it seems to result from a complex interplay of intrinsic and extrinsic factors. Ultraviolet radiation(UVR), ingestion of arsenic acid (medicine, pesticides), ionizing radiation, X-ray and grenz-ray exposure, topical nitrogen mustard administration, and thermal burns are considered as underlying extrinsic factors^1^. The intrinsic factors include gender, age, body mass index (BMI), fair skin, blond or red hair, light eye color, the tendency to sunburn, immunosuppression, and genetic predisposition (such as a family history of BCC)^2^. Therefore, restricting the intensity of UVR has been recommended as an effective way to prevent this cancer^3^. Besides, some dietary factors, such as an antioxidant and anti-inflammatory-rich diet, may also prevent skin cancer, including BCC^4^.

There appears to be an inverse association between obesity and non-melanoma skin cancer development^5^. Zhang Y et al. reported that being overweight and obese significantly reduced the risk of early-onset BCC^6^. In addition, a US cohort study found that increasing BMI was associated with decreased BCC risk in both men and women after adjusting for height and sun-related factors^7^.

Vitamin D is a crucial fat-soluble vitamin that performs several bioactive functions within the human body. It is primarily synthesized through exposure to ultraviolet radiation (UVR) in the skin. The 25-hydroxyvitamin D (25(OH)D) is a circulating metabolite of vitamin D that indicates vitamin D's status. Thus, the latest studies have shifted the focus on the relationship between vitamin D and BCC. According to Gordon-Thomson C’s research, vitamin D could prevent skin cancer by protecting keratinocytes from UV-mediated damage^8^. A Turkey hospital collected 7,396 cases from all age groups between October 2020 and 2021 and observed that vitamin D was a protective factor for BCC (*p* = 0.035)^9^. In contrast, several studies have reported an association between high levels of 25(OH)D and an increased risk of BCC^10^. Another prospective study involving women also yielded similar findings^11^. However, an earlier prospective cohort study that relied on dietary questionnaires to assess vitamin D intake showed no significant relationship between vitamin D and BCC^12^. Therefore, the causal effect between vitamin D and BCC remains unclear and needs to be further investigated.

Growing evidence has confirmed the association between vitamin D deficiency and obesity. For example, a prospective study's results in 2013 showed that weight loss could raise participants' serum 25(OH)D levels among obese women in a dose–response manner^13^. Furthermore, a meta-analysis concluded that the obese population was susceptible to suffering from vitamin D deficiency^14^. Given that possible relationship, exploring whether 25(OH)D takes part in and regulates the relationship between BMI and BCC is essential.

As the previous findings about obesity, 25(OH)D, and BCC are mostly based on observational studies and are easily interfered with by confounding factors and reverse causation. To address this issue, Mendelian randomization (MR) was used in our study. MR is an epidemiological technique that employs instrumental variable (IV) analysis to infer causal relationships between exposures and outcomes using genetic variants, particularly single-nucleotide polymorphisms (SNPs). Based on the principle that genotypes are randomly assorted at meiosis, the MR method can prevent reverse causation bias and limit confounders^15^. Therefore, compared to conventional epidemiological methods, MR studies are more convincing. Multivariable Mendelian randomization (MVMR) allows for equivalent analysis to mediation under the MR framework. Consequently, MVMR can be utilized to estimate mediation effects and the direct effect of each exposure on the outcome^16^.

In our study, we first performed a two-sample MR method to explore the causal effect of BMI and 25(OH)D levels on BCC. And given that 25(OH)D might play a mediation or confounding role in the BMI-BCC model, we further took multivariable MR analyses to evaluate the independence of BMI as a risk factor.

## Materials & methods

### Data source

The genome-wide association study (GWAS) summaries of BMI were obtained from a meta-analysis including up to 681,275 individuals of European ancestry with 2.3 million SNPs from the Genetic Investigation of Anthropometric Traits (GIANT) Consortium^17^. The IVs for 25(OH)D levels were derived from a GWAS study that identified 143 loci among 417,580 European ancestries by Revez JA’s team in 2020 ^18^. The GWAS summaries for BCC were from Adolphe C’s GWAS study performed among 17,416 European patients of BCC and 375,355 controls^19^.

The detailed information was mostly downloaded from the IEU Open GWAS database, containing 244,879,032,980 genetic associations from 42,334 GWAS summary datasets, and available for querying and downloading.

Since GWAS have ethical approval from their respective institutional review boards, incorporate informed consent from participants, and have rigorous quality control measures, this study did not require ehical approval.

### IV’s selection

In general, MR analysis needs to obey three assumptions: (1) the IVs should be significantly associated with the exposure^20^; (2) the IV variants are not associated with the confounding factors of exposure and outcome^21^; (3) the IVs should not affect the outcome except through exposure^22^. Based on these, we set a series of criteria to select SNPs in the two-sample MR method and MVMR analysis. First, to ensure the SNPs were significantly associated with BMI and 25(OH)D, we selected SNPs at P < 5 × 10–8 and those with linkage disequilibrium (LD) based on r2 > 0.001 and window size < 10,000 kb were excluded. Besides, we used F-statistic to calculate the strength of association between SNPs and exposure, where a score of > 10 indicated a sufficiently strong instrument^23^. Finally, the PhenoScanner database was used to examine whether SNPs were related to other potential risk factors, as these may lead to horizontal pleiotropy in the exposure-outcome association^24^. After finishing several procedures above, the remaining SNPs were considered as reliable IVs for MR analysis.

In the selection of IVs for MVMR analysis, we chose BMI as the main exposure and selected BMI-related SNPs under the condition that P < 5 × 10–8. After performing the harmonization step, we set a threshold of r2 > 0.001, window size < 10,000 kb as well, and the left SNPs performed MVMR analysis.

### Data analysis

Initially, we conducted a two-sample MR analysis, testing the total effects of BMI and 25(OH)D on BCC respectively. After that, considering BMI may affect the incidence of BCC by declining the serum level of 25(OH)D, we applied further MVMR to predict the direct effect of BMI on BCC and determine whether the level of 25(OH)D could mediate BMI.

We used inverse-variance weighted (IVW) as the primary method in two-sample MR analysis and regression-based IVWs in MVMR analysis. MR-Egger and Weighted median were also performed as the complementary methods. The IVW method combined ratio estimates of SNPs in an inverse variance-weighted manner by using a meta-analysis approach to evaluate the effect of risk factors on exposure^25^. MR-Egger regression is a tool to examine possible bias in meta-analysis and test the presence of pleiotropic effects of SNPs. The slope coefficient from MR-Egger regression can also be used to estimate the causal effect^26^. The weighted median is a novel method combining data on multiple genetic variants into a single causal estimate. The benefit of this method is even when more than 50% of IVs are invalid, the estimator remains consistent^27^. To test whether our results were reliable, we conducted sensitivity analyses. A Cochran Q test was carried out on IVW and MR-Egger to estimate heterogeneity, and the results were considered significant when the Q-P value < 0.05. The intercept test of MR-Egger stands for whether there are horizontal pleiotropic effects.

The statistical analyses in this study were carried out using R(version 4.0.3).

## Results

### The effect of BMI on basal cell carcinoma

**Figure 1:**
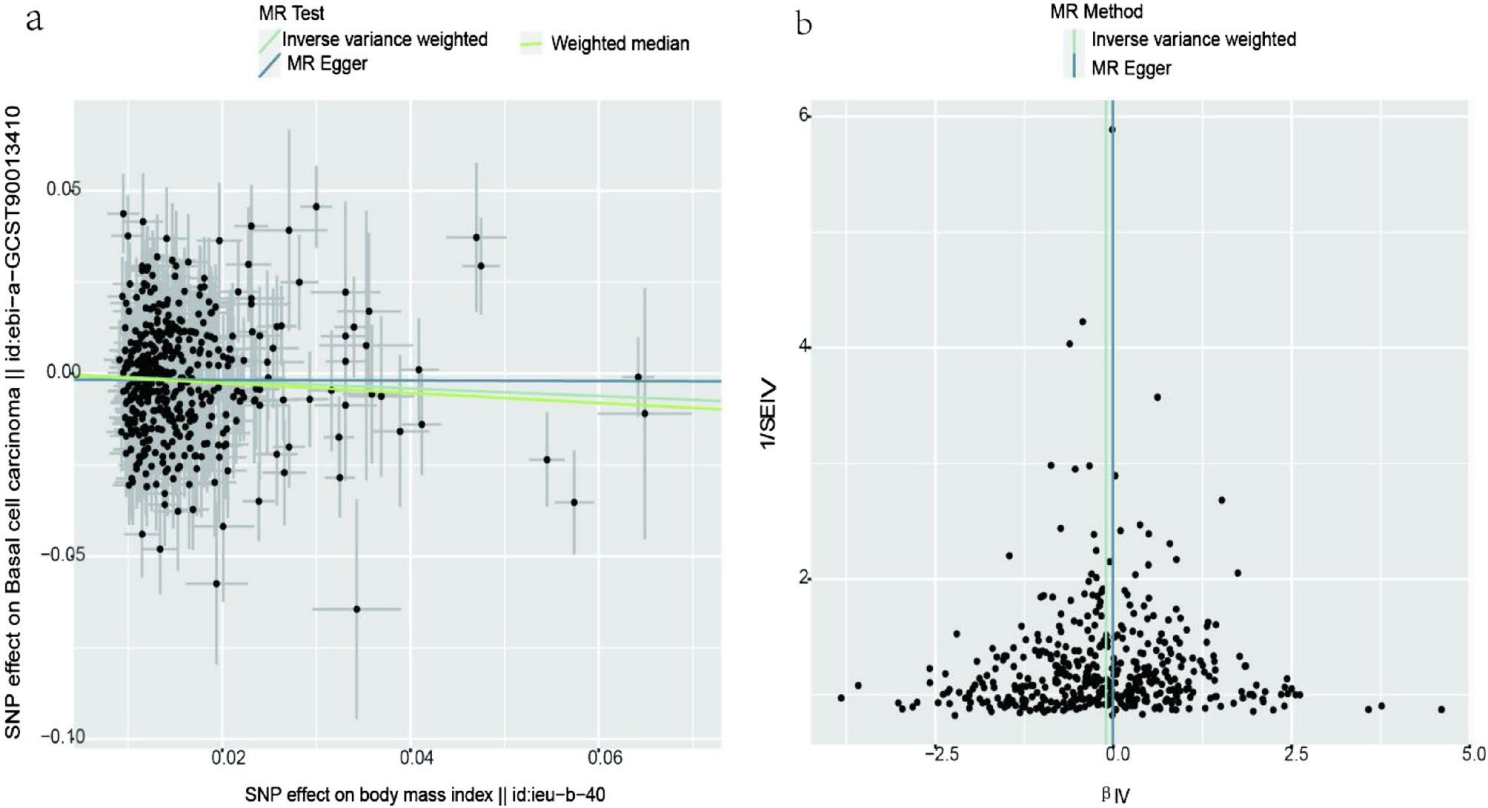
The Scatter plot and a funnel plot of the association between body mass index and basal cell carcinoma. (**a**) Scatter plot of the association between body mass index and basal cell carcinoma. The three methods applied in our study were all described. (**b**) A funnel plot was applied to detect the heterogeneity. MR, Mendelian randomization; IVW, Inverse variance weighted; SNP, Single nucleotide polymorphisms; IV, Instrumental variable.

443 SNPs were included in our analyses to estimate the association between BMI and BCC. The outcome showed a causal effect between BMI and BCC, and higher BMI may decrease morbidity from BCC. Due to the high heterogeneity (Q = 713, p = 4.49 × 10–15), we applied the IVW multiplicative random effects model, and the results still indicated that BMI played a protective role in the occurrence of BCC(Odds ratio(OR) = 0.90; 95%confidence interval (CI),[0.81,0.99]; *p* = 0.02). The weighted median results were similar to the IVW results(OR = 0.87; 95%CI,[0.74,1.00]; *p*  = 0.04). Despite lacking statistical significance, the results of the MR-Egger also showed the same direction as the other two methods (OR = 0.99; 95%CI,[0.76,1.22]; *p*  = 0.96)(Table 1)(Fig. 1a).

**Table 1:** Mendelian randomization and sensitive analyses of BMI and 25(OH)D on risk of BCC.

Exposures	outcomes	MR methods	OR	95%CI lower	95%CI upper	P value	Q test	Q_pval
BMI	BCC	IVW	0.90	0.81	0.99	0.02	713.33	4.49 × 10–15
		MR-Egger	0.99	0.76	1.22	0.96	712.02	4.53 × 10–15
		Weighted median	0.87	0.74	1.00	0.04		
25(OH)D	BCC	IVW	1.02	0.94	1.09	0.67	96.41	0.44
		MR-Egger	0.96	0.87	1.05	0.40	92.82	0.51
		Weighted median	0.99	0.89	1.10	0.82		

Abbreviations: BMI—Body mass index; 25(OH)D—25-hydroxy-vitamin D; BCC—Basal cell carcinoma; OR—Odds ratio; CI—Confidence interval; MR—Mendelian randomization; IVW—Inverse variance weighted.

MR-Egger intercept test showed no evidence of horizontal pleiotropy (MR-Egger interception:-0.002, p-value = 0.37). The funnel plots indicated there was no significant experimental bias (Fig. 1b). Based on the leave-one-out analysis, it was suggested that the exclusion of single nucleotide polymorphisms (SNPs) did not substantially alter the outcomes, which indicated that the results were vigorous (Fig. S1).

### The effect of 25-hydroxy-vitamin D levels on BCC

**Figure 2:**
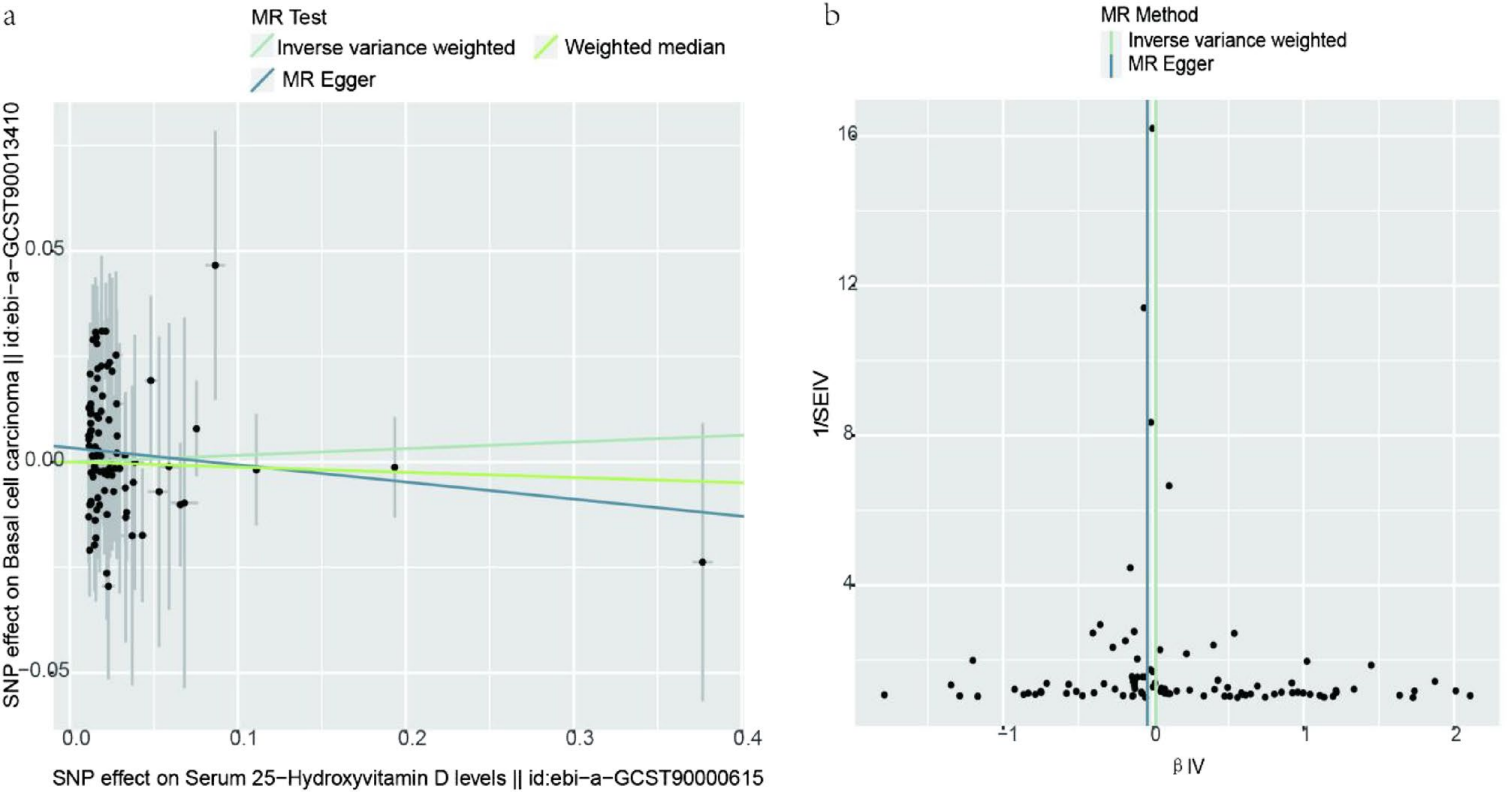
The Scatter plot and a funnel plot of the association between 25-hydroxy-vitamin D and basal cell carcinoma (**a**) Scatter plot of the association between 25-hydroxy-vitamin D and basal cell carcinoma. The three methods applied in our study were all described. (**b**) A funnel plot was applied to detect the heterogeneity. MR, Mendelian randomization; IVW, Inverse variance weighted; SNP, Single nucleotide polymorphisms; IV, Instrumental variable.

After the selection, we extracted 96 valid IVs to conduct MR analyses. The results of IVW did not support the causal relationship between serum 25(OH)D levels and BCC(IVW: OR = 1.02; 95%CI,[0.94,1.09],p-value = 0.67). We achieved similar results by using the other two methods (MR Egger: OR = 0.96,95%CI,[0.87,1.05],p-value = 0.40; Weighted median: OR = 0.99, 95%CI,[0.89,1.10],p-value = 0.82)(Table 1)(Fig. 2a). After calculating the intercept term of the MR-Egger, we found that there was no evidence of horizontal pleiotropy and heterogeneity between SNPs and outcome (Fig. 2b). The leave-one-out method showed that there was no single SNPs could influence the total result (*Fig. S2*).

### Multivariable MR analyses

The effects from the multivariable MR analysis revealed that the direct effect of BMI on BCC was still significant after we controlled SNPs standing for serum 25(OH)D level, and became slightly stronger than the total effect we calculated in two-sample MR(OR = 0.88; 95% CI,[− 0.22, − 0.03], p-value = 0.008). However, there is no evidence that 25(OH)D could affect BCC even though adjusting BMI(OR = 1.17, p-value = 0.45)(Table 2).

**Table 2:** Multivariable MR analyses of BMI and 25(OH)D on risk of BCC.

	MVMR method	Number of snps	OR	95%CI lower	95%CI upper	P value
BMI	IVW	414	0.88	0.79	0.98	0.008
25(OH)D	IVW	414	1.17	0.76	1.59	0.45

Abbreviations: BMI—Body mass index; 25(OH)D—25-hydroxy-vitamin D; BCC—Basal cell carcinoma; OR—Odds ratio; CI—Confidence interval; MVMR—Multivariable Mendelian randomization; IVW—Inverse variance weighted.

## Discussion

This study discovered that BMI might lower the risk of BCC at a genetic level. The MR method indicated this causal relationship between BMI and BCC, which agreed with previous retrospective observational studies and another two-sample MR research^28^. Although numerous epidemiological studies have reported that 25(OH)D plays a role in reducing various cancer morbidity, our results showed that vitamin D had no effect on BCC. When compared to two-sample MR research, MVMR allows for estimation of the different effects required for mediation analysis, which is performed to test whether these causal risk factors are independent of each other. In this study, the next-step MVMR results suggested that BMI was an independent factor for BCC, and BMI moderately increased incidence of BCC when the serum level of 25(OH)D changed.

A hospital-based case–control study in 1994 reported increasing body mass had an apparent protective effect on BCC^29^. So far, a large number of studies have investigated the potential pathogenesis under this association, and the two main hypotheses are: (a) the difference of cumulative UV radiation (UVR) between obese and normal-weight individuals; (b) the probable function of estrogens. The well-known risk factor for Non-Melanoma Skin Cancer (NMSC) including BCC and squamous cell carcinoma (SCC) is UVR. A previous study speculated that the relationship between higher BMI and lower risk of BCC might be because obese individuals were inclined to have lower levels of physical activity, which led to less cumulative lifetime UVR exposure and reduced the risk of BCC^30^. Pothiawala et al. also assumed that obesity might be a surrogate for lifetime sun exposure (e.g., time spent outdoors, chronic sun exposure) rather than a direct causal factor for BCC^5^. However, in Praestegaard C's cohort study, the inverse association between obesity and risk for NMSC could still be established after adjusting potentially confounding factors related to UVR susceptibility^31^.

Although the possible function of estrogens in this BMI-BCC relationship has been proposed recently, the relevant studies are few and the conclusion remains inconsistent. A 9-year follow-up observational study of the Women's Health Initiative found that overweight and obese women had a lower risk of NMSC than normal-weight women^32^. It seems that converting androgens to estrogens will become more active in obese individuals' adipose tissues^33^. Therefore, a higher BMI with elevated circulating estrogen levels will eventually lead to a lower risk of BCC^34^. This hypothesis has also been verified in mouse models^33^. However, this hypothesis is still controversial. For example, an experiment including 27, 347 postmenopausal women showed that neither using estrogen therapy alone nor combining it with progestin influenced the incidence of BCC^35^.

The association between 25 (OH)D and BCC has been discussed for decades, but the underlying mechanism is still unclear. Part of early studies speculated that there was an inverse relationship between 25 (OH)D concentrations in BCC risk or mortality^36,37^. The most popular mechanism behind this protective association is the mediate function of the vitamin D receptor (VDR). 1,25D restraints the hedgehog signaling pathway, which promotes the development of BCC, and inhibits the cell Proliferation of basal keratinocytes through VDR. In addition to that, vitamin D may also play role in reducing DNA damage in keratinocytes when exposed to UV radiation through several mechanisms, including the up-regulation of p53, inhibition of stress-activated kinases, and suppression of nitric oxide production. Therefore, UV-photoproduct accumulation is reduced and DNA adducts are consequently limited, which limits the production of DNA mutations that drive skin cancer^38^. But in some other research, high 25(OH)D will increase the risk of keratinocyte cancers (KC), including BCC^39^. It seems like the incidence of BCC would be increased with higher intakes of vitamin D^40^. According to a recent linear dose–response meta-analysis performed by Yahya Mahamat-Saleh et al., every increment of 30 nmol/L in 25(OH)D was linked to a 41% increase in BCC^41^. However, our MR analyses outcomes do not support any potential causal relationship between vitamin D on BCC, which is consistent with a previous two-sample MR study^42^. What causes this distinction between those observational studies above and our study might be the interference of some environmental confounders, such as exposure to sun and skin pigmentation and reverse causality sometimes. Besides this, the publication bias may also result in this difference.

Based on previous studies, obesity is often accompanied by a low deficiency of vitamin D. On one hand, with more serum, muscle, fat, and liver, the average level of vitamin D in obese people will decline accordingly^43^. On the other hand, impaired hepatic 25-hydroxylation in obese people also may lead to a lower level of 25(OH)D^44^. Besides this, some studies found that obese individuals' lower frequency outdoor activities caused less sunlight exposure when compared to normal-weight people, and that would injure the synthesis of vitamin D^45^. Considering that vitamin D might be a potential confounder between BMI and BCC, we took the MVMR to predict the direct effect of BMI.

To our knowledge, this is the first study that uses MVMR to investigate BMI, vitamin D, and BCC from a genetic perspective. The notable strengths of our MR method are as follows: Firstly, although considerable observational studies have suggested there is a causal relationship between BMI and BCC, MR methods have provided more reliable evidence. Genetic variants associated with risk factors are randomly assigned at birth. They have a long-term impact, so the results of MR are less susceptible to confounding factors and reverse causation. Secondly, we obtained large-scale summary genetic data from up to 681,275 individuals and identified robustly correlated SNPs as instrumental variables (IVs) with F statistics > 10. It ensured that our study had the adequate statistical power to explore the causal relationship between BMI, 25-hydroxyvitamin D (25(OH)D), and the risk of BCC. Thirdly, we employed the MVMR approach while adjusting for 25(OH)D to establish a relatively independent causal inference from BMI to BCC. Lastly, we conducted sensitivity analyses to test whether the data obtained from the MR methods was biased due to pleiotropy.

However, this study still has some limitations. Because (i): we can't filter and exclude all phenotypes related to BCC, such as the UV exposure's effect. (ii): the entirety of the exposure and outcome data utilized in this study were obtained from the European population. Hence, the causal inferences drawn from the European population may not be generalizable to other populations, such as Asians.

Collectively, our study has suggested that high BMI is an independent protective factor for BCC, and there is no causal relationship between 25 (OH)D levels and BCC. Overall, our results suggest obesity shouldn't be treated as a risk factor for BCC. Meanwhile, some interventions, such as increasing vitamin D supplements, are unlikely to influence BCC risk significantly.

### Supplementary Information

Supplementary Information 1.

Supplementary Information 2.

## Data Availability

GWAS summary statistics are obtained from public data resources platform. The data of body mass index can be accessed here [https://portals.broadinstitute.org/collaboration/giant/index.php/GIANT_consortium_data_files#WHR_GIANT_and_UK_BioBank_Meta-analysis_Summary_Statistics]. The GWAS summary statistics for the 25(OH)D is available here [https://gwas.mrcieu.ac.uk/datasets/ebi-a-GCST90000615/]. The BCC’s GWAS data can be obtained from [https://gwas.mrcieu.ac.uk/datasets/ebi-a-GCST90013410/]. The data that support the findings of this study are available within the article and its supplementary files.

## Abbreviations

BCC: Basal cell carcinoma
BMI: Body mass index
25(OH)D: 25-Hydroxy-vitamin D
GWAS: Genome-wide association study
MR: Mendelian randomization
SNP: Single nucleotide polymorphisms
IVW: Inverse variance weighted
MVMR: Multivariable Mendelian randomization
OR: Odds ratio
CI: Confidence interval
UVR: Ultraviolet radiation
IV: Instrumental variable
GIANT: Genetic investigation of anthropometric traits
LD: Linkage disequilibrium
NMSC: Non-melanoma skin cancer
VD: Vitamin D
VDR: Vitamin D receptor
