# Supplementary material for: Evaluating the effect of body mass index and 25-hydroxy-vitamin D level on basal cell carcinoma using Mendelian randomization
Source: Sci Rep. 2023 Oct 2;13:16552. doi: 10.1038/s41598-023-43926-w (PMC10545741; doi:10.1038/s41598-023-43926-w)
Supplement: Supplementary file 2 — Supplementary Information 2. [file 41598_2023_43926_MOESM2_ESM.docx]

Figure supplementary 1. The leave-one-out analysis about the effect of body mass index on basal cell carcinoma


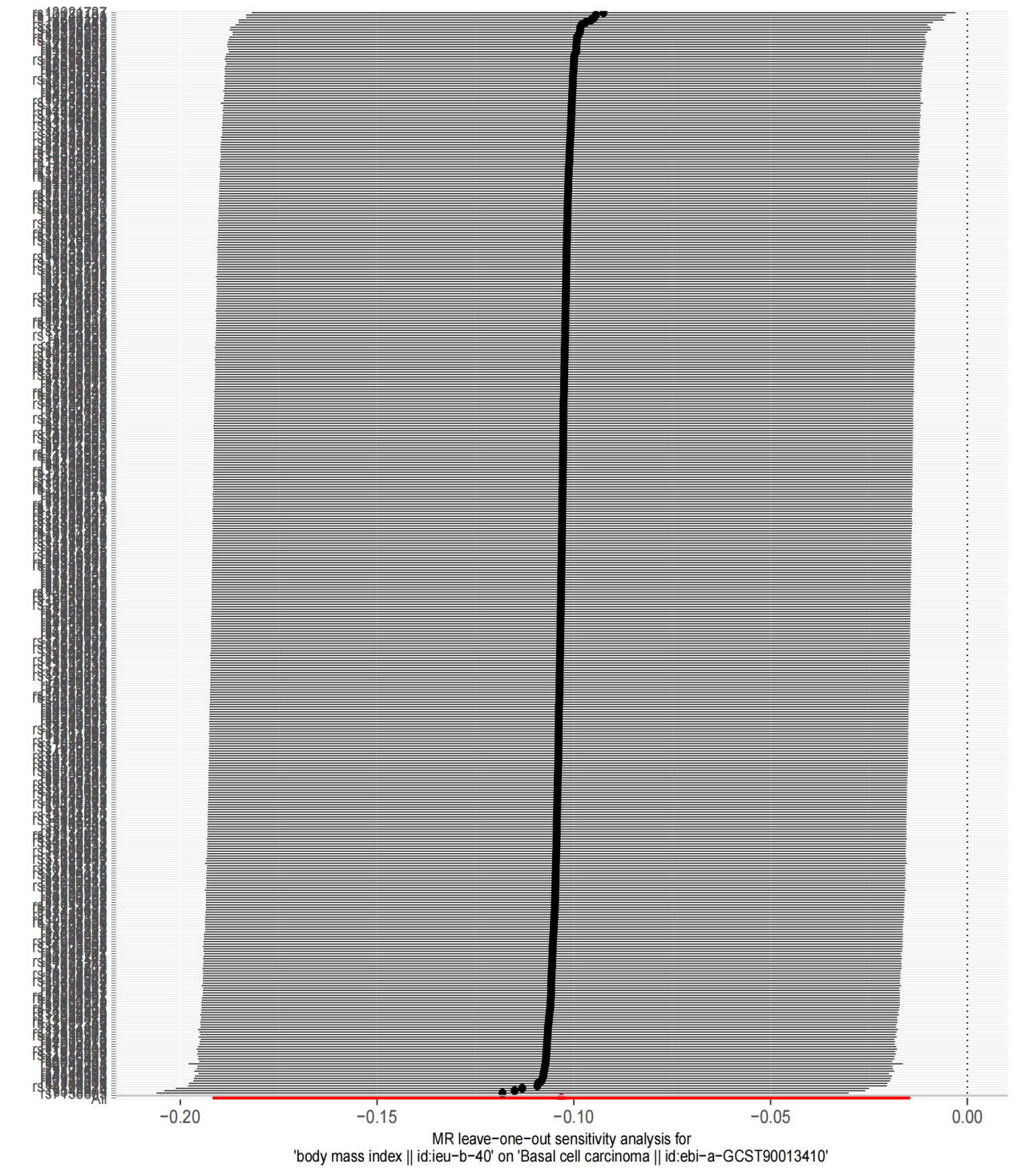


Abbreviations: MR—Mendelian randomization
